# Relationships among Trait Resilience, Virtues, Post-traumatic Stress Disorder, and Post-traumatic Growth

**DOI:** 10.1371/journal.pone.0125707

**Published:** 2015-05-01

**Authors:** Wenjie Duan, Pengfei Guo, Pei Gan

**Affiliations:** 1 Department of Applied Social Sciences, City University of Hong Kong, Hong Kong SAR, P. R. China; 2 Hospital (T. C. M.) Affiliated to Luzhou Medical College, Luzhou, Sichuan, P. R. China; 3 The First Affiliated Hospital of Chongqing Medical University, Chongqing, P. R. China; Technion—Israel Institute of Technology, ISRAEL

## Abstract

The present study aims to examine the relationship between trait resilience and virtues in the context of trauma. A total of 537 participants who attended the preliminary investigation and completed the Life Events Checklist were screened. Of these participants, 142 suffered from personal traumatic experiences in the past year; these individuals were qualified and invited to respond to online questionnaires to assess trait resilience, virtues (i.e., Conscientiousness, Vitality, and Relationship), post-traumatic stress disorder (PTSD) symptoms, and post-traumatic growth (PTG). The following questionnaires were used: Connor-Davidson Resilience Scale-Revised, Chinese Virtues Questionnaire, PTSD Checklist-Specific, and Post-traumatic Growth Inventory-Chinese. Only 95 participants who manifested self-reported PTSD symptoms and PTG were involved in the current analyses. Trauma was positively and significantly correlated with PTSD in the current sample. Results indicated that trait resilience was positively associated with virtues and PTG; by contrast, PTSD scores were negatively but not significantly related to most of these factors. The three virtues contributed to PTG to a greater extent than trait resilience in non-PTSD and PTSD groups. However, trait resilience remained a significant predictor in the PTSD group even when the three virtues were controlled. The relationship between trait resilience and PTG was moderated by PTSD type (non-PTSD group vs. PTSD group). Our results further suggested that trait resilience and virtues were conceptually related but functionally different constructs. Trait resilience and virtues are positively related; thus, these factors contributed variances to PTG in the context of trauma; however, trait resilience is only manifested when virtues are controlled and when individuals are diagnosed as PTSD. Furthermore, implications and limitations of this study are discussed.

## Introduction

Life-threatening events, such as earthquake, bereavement, and cancer, cause post-traumatic stress disorders (PTSD) and post-traumatic growth (PTG); PTG is indicated as positive changes and transcendences from traumatic experiences [1, 2]. Theoretical and empirical studies have suggested that personalities, traits, or qualities may be important predictors of health-related outcomes (such as PTG) in the context of trauma [3] because individuals with stronger positive traits are more likely to maximize internal (e.g., hopeful thinking and gratitude) and external (e.g., social support) resources to overcome adversities than other individuals. Previous studies demonstrated that virtues [4] and trait resilience [5] are positively related to PTG. However, these studies have not examined the relationship between trait resilience and virtues in the same traumatic context. Therefore, the present study aims to fill this gap.

Peterson and Seligman applied 24 human strengths and 6 virtues (i.e., wisdom and knowledge, courage, humanity, justice, temperance, and transcendence) to the same principle as diagnostic and statistical manual of mental disorders (DSM) to establish current classification, particularly values in action (VIA) classification of strengths [6]. For example, transcendence virtue refers to the ability of individuals to establish meaningful connection with other individuals and nature, as well as with the world; such connection includes strengths, such as appreciation of beauty and excellence, gratitude, hope, humor, and spirituality. Virtues are also defined as trait-like positive qualities that constitute basic factors influencing growth of humans [7, 8]; virtues facilitate individuals to pursue goals and ideals and to stimulate pleasure, flow (or a state of absorption by which one’s abilities are well matched to the demands), and other positive experiences [9]. Peterson and Seligman defined virtues as “a property of the whole person and the life that person leads” [6]. Studies have revealed that these positive components are positively related to psychological well-being [10] and enhanced mental health [11, 12] among diverse populations in western and eastern cultures. Study [4] examined the relationship between virtues and PTG by using 1,739 samples from different countries. In contrast to a theoretically proposed six-factor structure, a five-factor virtue structure, including interpersonal, fortitude, cognitive, transcendence, and temperance, based on data pool is established by principal component factor analysis. These five virtues are significantly and positively related to PTG [4].

Three approaches can be applied to define resilience based on previous studies [13]. In the first approach, resilience is defined as “recovery” from a low level after a traumatic event occurs to a normal level or baseline before a traumatic event happens. However, Bonanno [14] considered that recovery is not necessary to achieve resilience. Studies have also indicated that some trauma survivors may develop PTSD, whereas other survivors may not experience PTSD [15]. The daily functioning of survivors who have developed PTSD remains below normal level. Nevertheless, these survivors are likely resilient. In the second approach, resilience emerges or functions as a possible outcome of adversity [16]. Therefore, resilience is a process of rebounding and changing after an individual experiences trauma [5]. Based on this perspective, resilience is a context-dependent “reconfiguration” that overlaps with PTG [13]. To eliminate ambiguity between resilience and PTG, Agaibi and Wilson [17] recommended that resilience should be considered as a personality trait and PTG should be described as a mode of adjustment to trauma [18]. This recommendation is the third approach; in this approach, resilience is conceptualized as an idea of “resistance,” indicating the ability to resist negative change and remain stable [14]. The third definition hypothesizes that individuals with high trait resilience remain resilient before, during, and after individuals experience trauma. Bensimon [5] used a structural equation model to show that trait resilience positively predicts PTG among participants with various exposure levels; PTSD further mediates the relationship between trait resilience and PTG.

Fowers [7] and Robbins [19] suggested that trait resilience can be recognized as a virtue that can extend and enhance current virtue classifications in positive psychology [6]. The specific components included in both constructs in previous studies have been further examined, and similarities between trait resilience and virtues have been observed. For example, trait resilience contains a cluster of personalities, such as optimism, hope, self-improvement, self-regulation, self-enhancement, positive effect, and vitality, which help manage traumatic experiences [17, 18]; these personalities are character strengths in the VIA classification. Trait resilience likely overlaps with the virtue of courage, which refers to “emotional strengths that involve the exercise of will to accomplish goals in the face of opposition, external or internal” [6]. Previous study [20] investigated the relationship between psychological constructs among young adults and found that virtues and trait resilience exhibit a probable biological component.

Peterson, Park [4] and Hutchinson, Stuart [20] expressed concerns on virtue structure, which may provide contradicting conclusions. On the one hand, the five-factor virtue structure found by Peterson, Park [4] is inconsistent with the original six-virtue structure that they initially developed [6]. On the other hand, Hutchinson, Stuart [20] admitted that the six-factor virtue structure may not be appropriate in the South African context. The issue of virtue structure is not unique to these studies; various virtue structures, such as three-, four-, and five-factor structures, have been identified in different cultures [21]. The lack of cross-cultural functional equivalence and factorial invariance of a virtue system may partially explain this inconsistency [22, 23]. To explore cross-cultural and stable structure of virtues, Duan, Ho [21] applied the combined etic-emic approach to select 96 items that can be understood by Eastern and Western individuals from the original 240-item VIA inventory of strengths. Three virtues, namely, relationship (i.e., love, concern, and gratitude of a person toward others), vitality (i.e., curiosity and zest for creativity of an individual), and conscientiousness (i.e., an intrapersonal virtue that describes people who persist to achieve goals and exhibit self-control) [23], with construct invariance, high reliability, and theoretical meaning, have been identified by exploratory and confirmatory factor analyses [24]. These morally valued virtues manifest through cognition, emotion, motivation, volition, and action [6]. Subsequent studies demonstrated the virtues were positively related to life with satisfaction and flourishing [24–27], as well as negatively related to depression, anxiety, psychological distress, and pathology Internet use [24, 26, 28, 29]. Thus, the three-virtue structure is applied in the current study.

On the basis of literature review, we hypothesize that trait resilience and virtues are conceptually related constructs because both of these factors are traits or trait-like qualities that share the same probable biological component [20]. However, whether or not their functions in traumatic sample differ remains unclear. To the best of our knowledge, this study is the first to investigate the relationship between trait resilience and virtues in the same context of trauma. The relationship of these factors should be examined because health professionals likely consider their clients’ strengths [30]. Our results provide further insights into strength-based research and practical applications in trauma-related fields.

## Methods

### 1 Participants and Procedures

Data collection was divided into two stages. In the first stage, an invitation letter was published on the bulletin board system of several universities. Interested individuals were instructed to complete a 17-item Life Events Checklist (LEC) online. Only participants who personally experienced trauma were identified as qualified subjects of Stage 1 (Measures section); the qualified subjects were then invited to attend the next stage. In the second stage, the qualified participants were sent an E-mail with a link directing to web-based questionnaires. After stating their consent to participate (i.e., clicking the button to agree; thus, written consent was obtained from the participants before data were collected), these participants proceeded to answer the questionnaires. Only participants who reported both PTSD symptoms and PTG were included in the current study. Several methods were used in this study to control common method bias [31]. For instance, the web-based survey was designed to protect personal information and encourage subjects to present real reactions to sensitive questions; all items in the online survey system were arranged to appear randomly. Gosling, Vazire [32] and King, O’Rourke [33] considered that web-based data collection method is reliable and helpful in obtaining sensitive information and inaccessible populations. We applied these steps to ensure that all of the participants in the current study satisfied the A Criterion for PTSD in DSM-V [34]; in particular, a person was directly exposed to trauma, such as death, threatened death, actual or threatened serious injury, or actual or threatened sexual violence.

A total of 537 participants completed the LEC at Stage 1; among these participants, 26.44% (*n* = 142) personally experienced traumatic events. Among the 142 qualified individuals, 95 (66.90%) reported both symptoms of PTSD and PTG, whereas 47 did not exhibit symptoms of these conditions. A total of 95 participants who personally experienced traumatic events and reported both PTSD symptoms and PTG were included in the following analysis. Among the included participants, 76 (80.00%) are females and 19 (20.00%) are males. On the basis of previous studies [35], we did not examine gender difference because of unequal distribution. Among 95 students, 73 (76.80%) were 20 years old to 29 years old, whereas 22 were 18 years old to 20 years old. Furthermore, 77 of the participants (81.10%) are single and 18 (18.90%) are in a relationship. Demographic characteristics and frequency of traumas are shown in Table 1. The Human Subjects Ethics Committee of the Hospital (T. C. M) Affiliated to Luzhou Medical College approved this study and the consent procedure. Data were analyzed using Statistical Product and Service Solutions (SPSS) 20.0.

**Table 1:** Demographic and Sample Characteristics (*N* = 95).

Variables	*n*	%
**Gender**		
** Male**	19	20.00%
** Female**	76	80.00%
**Age**		
** Under 20 and above 18**	22	23.20%
** 20–29**	73	76.80%
**Types of trauma**		
** Physical assault (being attacked, hit, slapped, beaten up, kicked)**	50	52.60%
** Natural disaster (flood, hurricane, tornado, earthquake)**	34	35.80%
**Sudden, unexpected deaths of someone close to you**	26	27.40%
** Transportation accident (car accident, boat accident, train wreck, plane crash)**	16	16.80%
** Life-threatening illness or injury**	12	12.60%
** Fire or explosion**	10	10.50%
** Physically, sexually, or emotionally abused by someone close to you**	7	7.40%
** Serious accident at work, home, or during a recreational activity**	6	6.30%
** Assault with a weapon (being shot, stabbed, threatened with a knife, gun, bomb)**	4	4.20%
** Serious injury, harm, or death you caused to someone else**	4	4.20%
** Other unwanted or uncomfortable sexual experiences**	3	3.20%
** Exposure to toxic substances (dangerous chemicals, radiation)**	3	3.20%
** Sexual assault (attempted rape, forced or threatened to perform any type of sexual act)**	1	1.10%

Note: None of the participants identified four other kinds of trauma, including combat or exposure to a war zone (in the military or as a civilian), captivity (being kidnapped, abducted, held hostage, prisoner of war), severe human suffering, and sudden, violent death (homicide, suicide).

### 2 Measures

#### 2.1 LEC

LEC was used to screen individuals who suffered from traumatic experiences based on 17 different events [36], such as natural disaster (e.g., flood, hurricane, tornado, earthquake), life-threatening illness or injury, and fire or explosion. The participants were requested to rate each event based on their true experiences by using a five-point Likert scale (1 = happened to me, 2 = witnessed it, 3 = learned about it, 4 = not sure, 5 = does not apply). In this study, a time framework of “one year” was set in the instruction that required the participants to recall events that happened in the past year. Only the participants identified at least one traumatic event as “happened to me” (1) were considered as qualified samples; other responses were excluded in this study. The total score of the whole scale reflect the frequency of trauma; high scores indicated high frequency of experienced traumatic events.

#### 2.2 Connor-Davidson Resilience Scale-Revised (CD-RISC-R)

CD-RISC-R is a revised scale with 10 items and applied to assess individual trait resilience [37]. This scale exhibits a more stable structure, higher reliability, and better construct validity than the full CD-RISC [38]. Item samples include “able to adapt to change” and “tend to bounce back after illness or hardship.” In this study, the participants were instructed to rate each item on a five-point Likert scale ranging from 0 (not true at all) to 4 (true nearly all of the time). A high mean score corresponds to great resilience. The Chinese version of CD-RISC-R also shows good psychometric properties [39]. In the present study, Cronbach’s *α* value for the entire scale is 0.82.

#### 2.3 Chinese Virtues Questionnaire (CVQ)

A 96-item Chinese scale CVQ was used to assess the virtues [21, 24]. The respondents were requested to rate each item based on a five-point Likert scale ranging from 1 (very much unlike me) to 5 (very much like me). Item samples include “I can accept love from others” (relationship), “I like to think of new ways to do things” (vitality), and “I control my emotions” (conscientiousness). The mean scores of the three virtues (32-item relationship, 40-item vitality, and 24-item conscientiousness) were used. A high score reflects that the individual exhibits a high degree of the virtue. In this study, Cronbach’s *α* values of the three subscales are 0.84 (relationship), 0.82 (vitality), and 0.85 (conscientiousness).

#### 2.4 PTSD Checklist-Specific (PCL-S)

PTSD symptoms were quantified using the 17-item PCL-S in a five-point Likert scale. Respondents were required to rate their experience from 1 (not at all) to 5 (extremely) [40]. Item samples include “feeling very upset when something reminded you of the stressful experience” and “avoiding thinking about or talking about the stressful experience or avoiding having feelings related to it.” A high total score of the whole questionnaire indicates high degree of severity of PTSD symptoms. The participants who identified all items as “not at all” (1) were excluded in this study. In the current sample, Cronbach’s *α* of the whole scale is 0.89.

#### 2.5 Post-traumatic Growth Inventory-Chinese (PTGI-C)

PTG was quantified using the 15-item Chinese inventory scale PTGI-C [41]. Individuals indicated the extent to which they experienced changes as a result of crisis, ranging from 0 (not at all) to 5 (a very great degree). The PTGI-C is categorized using four factors, namely, self, spiritual, life orientation, and interpersonal, which correspond to the five-factor model of the PTGI’s original English version. Item samples included “I developed new interests” (self), “Knowing that I can count on people in times of trouble” (interpersonal), and “I’m more likely to try to change things which need changing” (life orientation). Previous studies confirmed the reliability and validity of the 15-item version [41]. The participants who identified all items as “not at all” (0) were excluded in this study. In the present sample, Cronbach’s *α* of the inventory was 0.80.

## Results

### 1 Descriptive Statistics and Correlation Analysis

The results of descriptive statistics and correlation analysis are shown in Table 2. Resilience was significantly and positively related to three virtues (*r* = 0.21 to 0.41, *p* < 0.01) and PTG (*r* = 0.53, *p* < 0.01). All of the three virtues (conscientiousness, relationship, and vitality) were positively associated with PTG (*r* = 0.48 to 0.59, *p* < 0.01), but only relationship was negatively associated with PTSD (*r* = −0.21, *p* < 0.05) and frequency of trauma (*r* = −0.28, *p* < 0.05). PTG was not significantly related to PTSD and frequency of trauma; however, PTSD was positively and significantly related to frequency of trauma (*r* = 0.27, *p* < 0.01). According to the criteria proposed by Blanchard, Jones-Alexander [42], scores of ≥44 can indicate PTSD. Thus, 36 participants (37.90%) were classified under the PTSD group and 59 participants (62.10%) were classified under the non-PTSD group. However, ANOVA revealed no significant differences among all of the variables between the two groups (PTSD group *vs*. non-PTSD group).

**Table 2:** Descriptive and Correlation Statistics of Trait Resilience, Virtues, PTSD, PTG, and Trauma Frequency (*N* = 95).

	Descriptive		Correlation					
	*M*	*SD*	1	2	3	4	5	6
**1 Resilience**	2.55	0.48	−					
**2 Conscientiousness**	3.50	0.63	0.41**	−				
**3 Relationship**	4.02	0.50	0.21*	0.51**	−			
**4 Vitality**	3.98	0.47	0.40**	0.35**	0.55**	−		
**5 PTSD**	2.46	0.73	−0.17	−0.09	−0.21*	−0.17	−	
**6 PTG**	4.12	0.44	0.53**	0.59**	0.48**	0.48**	−0.14	−
**7 Trauma Frequency**	1.85	1.34	0.06	−0.14	−0.28*	−0.07	0.27**	−0.01

* *p* < 0.05;

** *p* < 0.01.

### 2 Regression Analysis

Hierarchical regressions were performed to examine the predictive ability of trait resilience and virtues to PTG (set as dependent variable) in PTSD and non-PTSD groups. Two equations were constructed to further investigate the incremental validity of the two target variables. In the first equation, trait resilience was encoded in the first step and three virtues were encoded in the second step. In the second equation, three virtues were included in the first step and trait resilience was included in the second step. Table 2 shows the main results of regression analyses. In the non-PTSD group, conscientiousness contributed another 13% variance [0.38 to 0.51 = 0.13; Table 2, Equation (1)] to explain PTG after trait resilience was controlled; by contrast, trait resilience only contributed 3% [0.48 to 0.51 = 0.03; Table 2, Eq. (2)] after the virtues were controlled. In the PTSD group, the virtues additionally explained 43% variance when trait resilience was controlled [0.17 to 0.60 = 0.43; Table 2, Eq. (1)]. Likewise, trait resilience explained 14% variance when the virtues were controlled [0.46 to 0.60 = 0.14; Table 3, Eq. (2)]. Therefore, virtues likely exhibit a more powerful predictive ability than trait resilience in non-PTSD and PTSD groups; trait resilience contributed more variances of PTG in the PTSD group (14%) than in the non-PTSD group (3%). In the non-PTSD group, trait resilience was also not a significant predictor after the virtues were included in the equation [Table 3, Eq. (1)]; by contrast, trait resilience remained significant in the PTSD group. These results suggested that the severity of symptoms of PTSD might moderate the relationship between trait resilience and PTG.

**Table 3:** Regression of Trait Resilience and the Three Virtues on PTG in Different Subgroups (*N* = 95).

	Non-PTSD Group (*n* = 59)				PTSD Group (*n* = 36)			
	***R*** ^***2***^	***F***	***Beta***	***t***	***R*** ^***2***^	***F***	***Beta***	***t***
**Equation One**								
**Step 1**	0.38	35.05***			0.17	6.79*		
** Resilience**			0.62	5.92***			0.41	2.61*
**Step 2**	0.51	14.21***			0.60	11.466***		
** Resilience**			0.25	1.84			0.41	3.29**
** Conscientiousness**			0.40	3.36***			−0.09	−0.48
** Relationship**			0.08	0.74			0.73	3.30**
** Vitality**			0.18	1.45			−0.01	−0.05
	Δ*R* ^*2*^ = 0.13**				Δ*R* ^*2*^ = 0.43***			
**Equation Two**								
**Step 1**	0.48	17.08***			0.46	8.94***		
** Conscientiousness**			0.51	5.05***			0.12	0.59
** Relationship**			0.13	1.11			0.48	2.04*
** Vitality**			0.26	2.21*			0.14	0.81
**Step 2**	0.51	14.21***			0.60	11.466***		
** Conscientiousness**			0.40	3.36***			−0.09	−0.48
** Relationship**			0.08	0.74			0.73	3.30**
** Vitality**			0.18	1.45			−0.01	−0.05
** Resilience**			0.25	1.84			0.41	3.29**
	Δ*R* ^*2*^ = 0.03				Δ*R* ^*2*^ = 0.14**			

* *p* < 0.05;

** *p* < 0.01;

*** *p* < 0.001.

### 3 Moderation Effect Analysis

**Fig 1:**
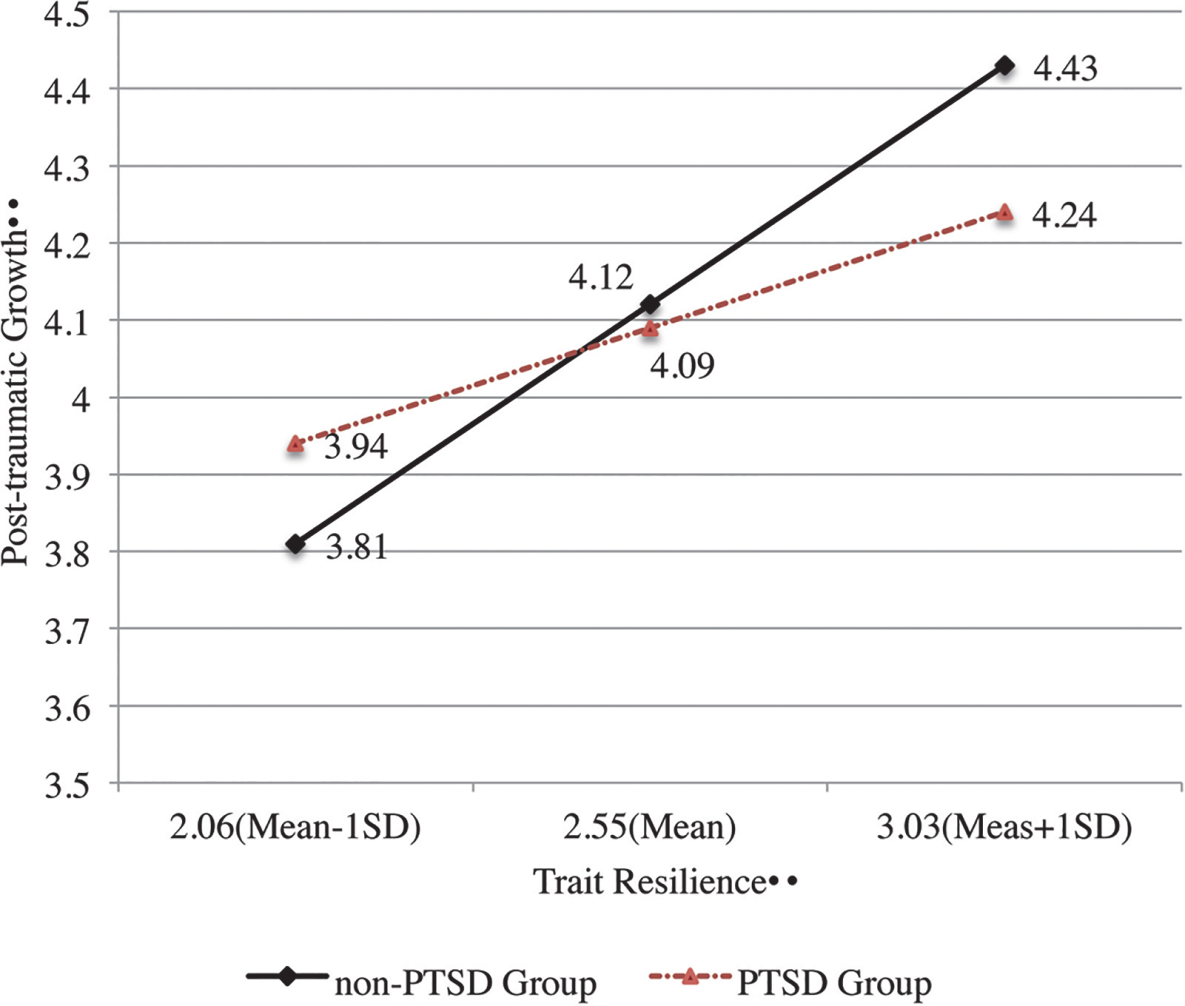
Interaction Effect between Trait Resilience and PTSD Groups on PTG.

The moderation effect was examined using Model 1 in PROCESS [43]. Based on the results, trait resilience was set as predictor (X), PTG was set as outcome (Y), and PTSD group was set as moderator (M). The full model was significant: *R* ^*2*^ = 0.315, MSE = 0.139, F (3,91) = 18.899, *p* < 0.001. Table 4 indicates that the interaction variable (Resilience × PTSD Group) and the slopes of the two groups were significant; therefore, the PTSD group can moderate the relationship between trait resilience and PTG. Fig 1 further shows that the slope of the non-PTSD group was higher than that of the PTSD group; this result implied that the promoting role of trait resilience in the PTG group likely became weaker than that in the PTSD group.

**Table 4:** Moderation Effect Analysis of PTSD Group Based on PROCESS (*N* = 95).

	*Coeff*.	*SE*	*t*	*p*	LLCI	ULCI
**Overall Model**						
** (Constant)**	2.492	0.278	8.964	<0.001	1.940	3.045
** PTSD Group (M)**	0.811	0.363	2.235	0.028	0.090	1.531
** Resilience (X)**	0.641	0.103	6.229	<0.001	0.437	0.846
** Resilience × PTSD Group (X × M)**	−0.333	0.146	−2.282	0.025	−0.622	−0.043
**Conditional Effects**						
** Non-PTSD Group**	0.641	0.103	6.229	<0.001	0.437	0.846
** PTSD Group**	0.308	0.103	2.986	0.004	0.103	0.514

Note: PTSD group refers to the dichotomous variable, namely, the non-PTSD group (0) and the PTSD group (1); dependent variable is post-traumatic growth (Y).

## Discussion

The present study aims to explore the relationship between trait resilience and three virtues based on a sample with personal trauma in one year and on self-reported PTSD and PTG. These findings indicated that virtues and trait resilience were conceptually related but functionally different constructs. Trait resilience and virtues were positively related and contributed variances to PTG in the context of trauma; however, trait resilience manifested after the virtues were controlled when individuals were diagnosed as PTSD.

The current traumatic sample experienced a wide range of different traumatic experiences, such as natural disaster, physical assault, transportation accident, and sudden, unexpected deaths of a close relative or individual. Previous studies revealed that the increased frequencies of traumas lead to increased risks of PTSD symptoms [44]; likewise, the present study reveals the positive relations between the frequency of trauma and the severity of PTSD symptoms. Hagenaars, Fisch [45] indicated that multiple trauma individuals among a clinical sample usually report more dissociation, guilt, and interpersonal sensitivity than those who only experienced single trauma. However, these associations are not observed after PTSD severity is controlled [45]. These findings suggested that differences between clinical [45] and non-clinical (e.g., the current study) samples may result in inconsistent conclusions. Time point is also a factor that may affect such a relationship. Ogle, Rubin [46] found that events with similar greater frequency that occur early in life can yield more severe PTSD symptoms compared with those that occur later in life. Thus, frequency, time, and type of events should be carefully considered in future trauma-related studies.

In this study, a high level of trait resilience and three virtues were positively associated with PTG but negatively and not significantly associated with PTSD. Trait resilience and system of virtues share many common personality characteristics (hopeful, optimistic, and self-reliant). Thus, trait resilience is positively related to virtues; this finding is consistent with that in previous studies [46]. Regarding the relationship between virtues and PTG, Hutchinson, Stuart [20] suggested that a number of strengths and virtues in the current VIA classification correspond to the dimensions of PTG. Therefore, these “theoretically related strengths,” as presented in the current study, should be positively related to PTG. For instance, kindness and love are theoretically related strengths of improved relationships with other individuals; religiousness is the related strength of spiritual development. Peterson, Park [4] compared the scores of personal strengths of US citizens before and after the 9/11 terrorist attacks. The authors proposed that strengths are possibly enhanced after individuals experience traumatic events [47]. These findings may be due to measurement overlap. In the Methods section, the item used to assess vitality virtue “I like to think of new ways to do things” was similar to the item used to assess PTG “I developed new interests;” both of these items reflected the meaning of creativity and curiosity. On the basis of these findings and reasons, a person may think that the boundary between virtues and PTG remains unclear and conceptually overlapping. As such, we recommend that the approach of distinguishing between trait resilience and PTG should be applied to respond to trauma; therefore, virtues as positive traits are manifested before, during, and after traumatic events occur, whereas PTG as a mode of adjustment to trauma is exhibited only after traumatic events occur.

The present study further indicates that virtues contribute to PTG to a greater extent than trait resilience in both non-PTSD and PTSD groups; however, trait resilience and the virtue of relationship remains a significant predictor in PTSD sample even when the other virtues are controlled. Regression-based PROCESS analysis demonstrated that the dichotomous PTSD group (non-PTSD group *vs*. PTSD group) moderated the relationship between trait resilience and PTG. Studies have considered PTG as unrealistic beliefs and positive illusions, whereas other studies have considered PTG as a positive identity change [18]. If PTG corresponds to unrealistic optimism to manage encountered adversity [48], resilient individuals should not have to experience such illusions [49]; this outcome affirms the negative relationship between resilience and PTG. However, the present data do not support this idea. On the contrary, trait resilience is positively related to and significantly predicts PTG even in the PTSD group. The PTSD group also shows the relationship between trait resilience and PTG. Researchers argued that only individuals with symptoms of PTSD can develop PTG [5]. This finding likely supports our assumption. The current study implies that the “true” PTG requires PTSD after adversity occurs, and trait resilience is necessary to the “true” PTG. Dekel, Mandl [50] suggested that PTG may be an actual outcome of the response to threat and trauma. In this regard, virtues are functionally different from trait resilience.

Three virtues contributed additional variances to PTG after trait resilience was controlled. These virtues also exhibit specific functions in samples with or without PTSD diagnosis. Duan and Guo [51] found that vitality uniquely contributes variances to stress-related PTG in an indirect trauma sample. The result was validated by another undergraduate sample. Individuals with high vitality often perceive less stress from minor events, which lead to less psychological symptoms [28]. These findings suggested that vitality might be a protective factor under low-pressure situations rather than trauma or high-pressure situations. One intervention study found that undergraduates with high vitality virtue are more willing to express their unsatisfied thinking and negative ideas related to daily stress to improve mental health; this approach partly shows the protective role of vitality [52, 53].

With regard to the direct trauma sample without PTSD, previous study found that relationship and conscientiousness significantly contribute to PTG, whereas only conscientiousness uniquely explains PTG in the direct trauma sample with PTSD [51]. A 30-year longitudinal study further indicated that self-controllability, which is manifested by the conscientiousness virtue, uniquely predicts PTG when PTSD is controlled [50]. However, these findings were slightly different from the current one; in particular, vitality and conscientiousness significantly contributed to PTG in the non-PTSD group; by contrast, only relationship uniquely contributed to PTG in the PTSD group. According to personality-event congruence hypothesis [54], personality-related vulnerabilities to specific stressors are sensitive to their correspondingly different stressful life events. Thus, different types of traumatic event can cause such inconsistency; thus, the current sample experiences a wide range of different traumas, whereas the previous sample almost experiences earthquake-related traumas [51]. Another possible explanation is that previous studies [50, 51] did not analyze trait resilience. Although the results are slightly different, the protective function of virtues under stressful situations can be expected. For instance, a meta-analysis has indicated that social support is a very important contributor to PTG in 103 studies [55]. Individuals with stronger relationship virtue are more likely to maximize external resources (e.g., social support) to overcome adversities caused by trauma. A few studies have examined the role of virtues in the context of trauma; as a result, conclusions cannot be drawn from limited studies. Nevertheless, we can conclude that trait resilience is probably a more stable and reliable factor affecting PTG than virtues in the context of trauma; by comparison, virtues are more sensitive to different stressful situations than trait resilience.

Some limitations of the current study should be discussed. First, event types may be an important influencing factor, which was not examined in the current study. Previous studies demonstrated that associations among PTG, PTSD, and related factors may depend on event types [51]. However, the researchers recommend that further studies should be conducted before any conclusions can be drawn because of the limited sample size of the present study. Second, the current study only involved undergraduate students, who seldom experience specific traumatic events, such as cancer and bereavement; these students might not show an in-depth understanding of growth after trauma. Community sample and more psychological outcomes should be examined in future studies. Third, causal relationship cannot be determined from the current cross-sectional design. Hence, future studies should adopt a longitudinal design and a large sample size to identify changes in trait resilience and virtues before and after traumatic events occur. At concept and measurement level, resilience, virtue, and PTG likely overlap or show multiple conceptualizations, such as outcome, state, or trait. For instance, Dekel, Mandl [50] investigated the predictors of PTG and PTSD and suggested that PTG and PTSD possibly exhibit a shared psychological component. Therefore, future research should be conducted to distinguish and provide precise definition and measurements.

## Supporting Information

S1 Dataset (ZIP)
